# 血管内外周T细胞淋巴瘤的临床病理特征

**DOI:** 10.3760/cma.j.issn.0253-2727.2021.07.009

**Published:** 2021-07

**Authors:** 雪 管, 亦宸 杨, 昱 祁, 文辰 巩, 晓莹 徐, 雅蕾 王, 玉虹 郭, 烨 罗, 琳 孙, 凯 付, 斌 孟

**Affiliations:** 1 天津医科大学肿瘤医院病理科，国家肿瘤临床医学研究中心，天津市肿瘤防治重点实验室，天津市恶性肿瘤临床医学研究中心，天津医科大学肿瘤医院中美淋巴血液肿瘤诊治中心 300060 Department of Pathology, Tianjin Medical University Cancer Institute and Hospital, National Clinical Research Center for Cancer; Key Laboratory of Cancer Prevention and Therapy, Tianjin 300060; Tianjin's Clinical Research Center of Cancer, Tianjin 300060, China; 2 天津医科大学肿瘤医院中美淋巴血液肿瘤诊治中心，美国罗斯威尔帕克癌症研究所病理科 Department of Pathology, Roswell Park Cancer Institute, Buffalo, New York

**Keywords:** 外周T细胞淋巴瘤，非特指型, 血管内淋巴瘤, 淋巴结, 病理诊断, Peripheral T-cell lymphoma, NOS, Intravascular lymphoma, Lymph node, Pathologic diagnosis

## Abstract

**目的:**

总结血管内T细胞和NK细胞淋巴瘤的临床病理特点，增加对此类疾病的认识，以减少漏诊、误诊。

**方法:**

回顾性分析1例原发于淋巴结内的血管内外周T细胞淋巴瘤，非特指型（IVPTCL，NOS）患者的临床及病理学特点，并复习相关文献资料。

**结果:**

患者，男性，66岁，PET-CT示全身多发淋巴结肿大，活检示正常淋巴结结构消失，淋巴滤泡部分破坏。高倍镜下显示大量血管弥漫增生、扩张，其内见异型淋巴样细胞团局限生长于管腔内，部分浸润大血管壁，细胞中等偏大，胞质中等，核不规则，可见单个或多个核仁，染色质呈凝聚状，部分空亮，核分裂象可见。肿瘤细胞表达CD3、CD43、CD8、GrB、TIA-1及穿孔素等；原位杂交EBV-EBER（−）；TCR基因重排检测（+）。予CHOP联合西达本胺方案化疗，患者不足2个月因感染、心肺功能衰竭死亡。检索相关文献，收集到有明确分型的血管内T细胞和NK细胞淋巴瘤56例，其中鼻型结外NK/T细胞淋巴瘤47例（男性27例，女性20例），间变性大细胞淋巴瘤8例（男性3例，女性5例），仅有1例为原发于脑组织的IVPTCL，NOS。本例为第2例报道的IVPTCL，NOS，且为第1例原发于淋巴结内的病例报道。

**结论:**

血管内T细胞和NK细胞淋巴瘤为高度侵袭性疾病，目前尚无有效治疗方案。此病累及淋巴结的报道少，尚需积累更多病例进一步研究。

## Abstract (en)

**Objective:**

To summarize the clinical and pathological features of intravascular NK and T cell lymphoma for better understanding of such disease to reduce misdiagnosis and miss-diagnosis.

**Methods:**

Clinical and pathological features were analyzed retrospectively in one case of intravascular peripheral T-cell lymphoma, not otherwise specified (IVPTCL, NOS), with literatures review.

**Results:**

The case presented in this study was a 66-year-old man. PET/CT scan showed multiple lymph nodes enlargement throughout the body. Normal lymph node structure could not be observed by tissue biopsy, while lymph follicles were partially disrupted. High-power light microscope revealed a large number of blood vessels with diffuse proliferation and dilation, where atypical lymphoid cell mass was restricted in the lumen and partially infiltrated the large blood vessel wall. These tumor cells were medium to large with moderate cytoplasm. The nucleus was irregular, single or multiple nucleoli could be seen, chromatin was condensed, some were empty and bright, and mitotic figures could be seen. Immunohistochemical staining showed that the neoplastic cells were positive for expression of CD3, CD43, CD8, GrB, TIA-1 and perforin. EBER in situ hybridization result was negative. Polymerase chain reaction test identified a clonal gene rearrangement of T-cell receptor γ. The patient was treated with CHOP in combination with chidamide, but died of infection and cardiopulmonary failure within 2 months. 56 cases of intravascular NK/T cell lymphoma with definite classification were collected from relevant literatures, including 47 cases with nasal type of extranodal NK/T cell lymphoma (27 were male and 20 were female), 8 cases with anaplastic large cell lymphoma (3 males and 5 females), and only one case with de nova IVPTCL, NOS in brain. We report the second case of IVPTCL,NOS, and notably originated from lymph node for the first time.

**Conclusion:**

Intravascular NK/T cell lymphoma is a highly aggressive disease with no effective treatment at present. Involvement of Lymph node has rarely been reported, and further studies on more cases are necessary.

血管内淋巴瘤（intravascular cell lymphoma，IVCL）属于非霍奇金淋巴瘤的少见类型，其组织病理学特点为淋巴样肿瘤细胞局限生长于血管腔内，主要以B细胞来源为主，少数为T细胞和NK细胞来源。近年来有关T细胞和NK细胞来源的IVCL病例报道越来越多，但大多为EBV阳性的鼻型结外NK/T细胞淋巴瘤，少数为ALK阳性或阴性的间变性大细胞淋巴瘤（ALCL）。本文报告1例罕见原发于淋巴结的血管内外周T细胞淋巴瘤，非特指型（IVPTCL，NOS），并复习相关文献，以提高对此类疾病的认识。

## 病例与方法

1. 病例资料：患者，男性，66岁。既往心肌梗死、肺结核及甲状腺功能低下病史，长期服用抗血小板药物。患者于两个月前出现午后低热，体温最高37.8 °C，伴盗汗、咳嗽、咳痰及乏力。就诊于外院，血常规示：PLT 18×10^9^/L；超声示脾大；胸部CT示双肺肺气肿伴多发肺大疱，纵隔淋巴结肿大，除外结核病；骨髓活检示造血功能低下（具体不详）。嘱停用抗血小板药物，血小板恢复后出院。出院后患者仍有发热、盗汗，行PET-CT检查示：①双侧腮腺区、颈后皮下、颈深部、颌下、颏下、下颈部、锁骨区及腋下多发结节，大者约2.0 cm×1.1 cm，CT值38 Hu，边界欠清，PET-CT示不同程度放射性浓聚，SUV值5.1；纵隔内气管右侧旁、腔静脉后、主肺窗、左肺动脉旁、右心膈角、内乳区及双肺门多发结节，大者约2.0 cm×1.1 cm，CT值50 Hu，边界欠清，PET显像可见放射性浓聚，SUV值3.5；胃左动脉区、肝门区、门-腔静脉间多发结节，大者约2.1 cm×1.0 cm，CT值34 Hu，边界欠清，PET显示异常放射性浓聚，SUV值3.5；双侧髂脉管区及腹股沟多发结节，大者约1.5 cm×0.9 cm，CT值34 Hu，边界欠清，PET显示不同程度放射性浓聚，SUV值3.6。②脾大，PET显像可见较高放射性浓聚，SUV值4.4。③鼻咽部未见放射性异常浓聚影。以上①、②考虑淋巴增殖性疾病，恶性淋巴瘤可能性大，建议淋巴结活检。患者为求进一步诊疗于2019年4月26日就诊于天津医科大学肿瘤医院。入院后查体示皮肤无皮疹、红斑，浅表淋巴结未触及明显肿大。血常规示RBC 3.79×10^12^/L［参考值（4.3～5.8）×10^12^/L］，WBC 4.61×10^9^/L［参考值（3.5～9.5）×10^9^/L］，PLT 114×10^9^/L［参考值（125～350）×10^9^/L］。于全麻下行腹股沟淋巴结切除并做病理检查。

2. 方法：手术切除标本经4％中性甲醛溶液固定，常规脱水、透明、石蜡包埋、切片、HE染色，光镜观察。免疫组化染色采用EnVision二步法，使用Roche Benchmark XT全自动免疫组化染色仪，所用抗体CK-pan、CD56、穿孔素、TDT、PD-1、CD4、CD7、PAX5、CD34、CD99、CD30、TIA-1、CD21、CD31、CD2、GrB、CD10、MPO、D2-40购自北京中杉金桥生物技术有限公司，Ki-67、CD3、CD20、CD8、CD5、CD43、CD79a、LCA购自罗氏诊断产品（上海）有限公司，操作步骤严格按照试剂盒说明书进行。EB病毒（EBV）编码小RNA（EBER）原位杂交试剂盒购自罗氏诊断产品（上海）有限公司，按产品说明书操作。TCRγ、β基因重排采用BIOMED-2引物系统，按试剂盒说明书操作进行。

3. 文献复习：检索中、英文文献中报道的血管内T细胞和NK细胞来源的淋巴瘤，并对其临床及病理特点进行汇总分析、比较。

## 结果

1. 病理结果：巨检：腹股沟切除物见淋巴结样肿物数枚，最大者3.3 cm×1.7 cm×1.2 cm，最小者直径0.5 cm，包膜完整，切面实性，呈灰红、灰黄，质地中等。镜检：低倍镜下正常淋巴结结构消失，淋巴滤泡部分破坏，部分极性存在，套区可见，T区增宽。高倍镜下见大量血管弥漫增生、扩张，其内见异型淋巴样细胞团局限生长于管腔内，部分浸润大血管壁，细胞中等偏大，胞质中等，细胞核不规则，可见单个或多个核仁，染色质呈凝聚状，部分空亮，核分裂象可见（图1）。免疫表型：肿瘤细胞呈T细胞不全表型，并表达CD8及细胞毒性分子。免疫组化示：LCA、CD3（图2A）、CD43、CD8（图2B）、细胞毒性分子Granzyme B（图2C）、TIA-1（图2D）、穿孔素阳性；CD2部分阳性；CD31、CD34示肿瘤细胞所在的血管内皮阳性（图2E、F），而D2-40阴性；CD4、CD5、CD7（图2G）、CD20、CD56、CD79a、PAX5、CD30、PD-1及TDT均阴性；Ki-67显示肿瘤细胞具有较高的增殖活性（图2H）。分子原位杂交示EBV-EBER阴性。TCR基因重排检测示T细胞单克隆性增生（图3）。

**图1:**
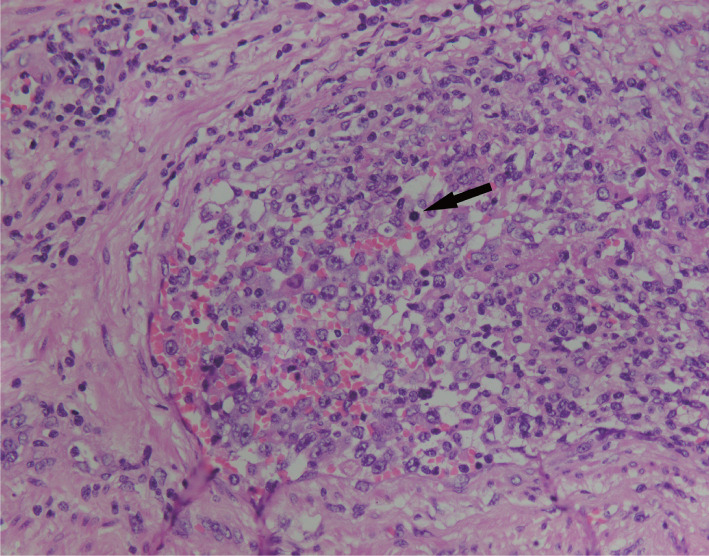
患者淋巴结切检组织病理HE染色（×400）可见核分裂象（箭头所指）

**图2:**
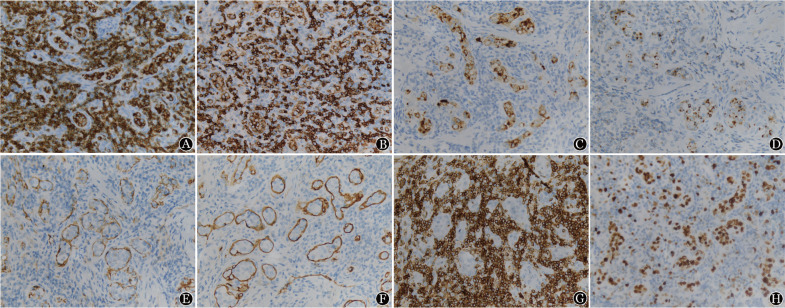
患者淋巴结切检组织免疫组化染色结果（×400） A：CD3免疫组织化学染色，肿瘤细胞（+）；B：CD8免疫组织化学染色，肿瘤细胞（+）；C：Granzyme B免疫组织化学染色，肿瘤细胞（+）；D：TIA-1免疫组织化学染色，肿瘤细胞（+）；E：淋巴结CD31免疫组织化学染色；F：淋巴结CD34免疫组织化学染色；G：CD7免疫组织化学染色，肿瘤细胞（−）；H：Ki-67免疫组织化学染色

**图3:**
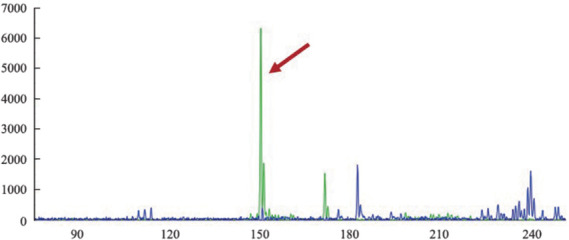
患者TCR基因重排检测显示TCRγ-A管中呈现克隆峰（箭头所指）

2. 病理诊断：（左腹股沟淋巴结）结合形态学、免疫表型及分子检测最终病理诊断为外周T细胞淋巴瘤，非特指型，因肿瘤细胞主要位于血管内，故诊断为IVPTCL，NOS（该类型尚未被WHO分类命名）。

3. 治疗及预后：予患者2个周期的CHOP+西达本胺治疗，具体方案：长春地辛4 mg第1天，脂质体阿霉素50 mg第1天，环磷酰胺1 g第1天，泼尼松80 mg第1～5天，西达本胺30 mg，每周2次。因患者既往有心肌梗死病史，于第2个周期化疗后再次出现低热，后病情逐渐恶化，于确诊后不到2个月死于心功能衰竭、肺功能衰竭、肺感染。

4. 文献复习：目前有近百例关于血管内T细胞和NK细胞淋巴瘤的报道，其中免疫表型完整、能够明确分型者56例，其余30余例为早期报道，免疫表型不完整，仅能证实为T细胞来源[1]–[2]。目前关于血管内T细胞和NK细胞淋巴瘤的分型诊断尚无明确诊断标准，根据WHO关于成熟T细胞和NK细胞肿瘤的诊断标准，大致分为以下几类：血管内NK/T细胞淋巴瘤（鼻型）（IVNKTL）、ALK^−^血管内ALCL（ALK^−^ IVALCL）、ALK^+^血管内ALCL（ALK^+^ IVALCL）、IVPTCL，NOS，还有数例累及皮肤的血管内CD30^+^T淋巴细胞增殖性疾病的报道[3]。本文报道1例原发于淋巴结内的IVPTCL，NOS病例，既往仅有1例原发于脑组织的IVPTCL，NOS病例报道，患者为62岁老年女性，治疗1周后死亡[4]。本例为第2例IVPTCL，NOS病例报道，且为第1例原发于淋巴结内的病例报道。患者为老年男性，发病伴随B症状，全身多发肿大淋巴结。两例患者均表达CD3、CD8及细胞毒性分子，均不表达CD4、CD5、CD7、CD56，原位杂交EBV-EBER阴性。关于血管内T细胞或NK细胞淋巴瘤的报道主要为NK/T细胞表型的病例[1],[5]–[36]（表1），其次为ALK^+^ IVALCL、ALK^−^ IVALCL[37]–[44]（表2）。

**表1:** 47例血管内NK/T细胞淋巴瘤患者的临床病理资料

例号	文献来源	年龄（岁）	性别	受累部位	骨髓受累	免疫组化及原位杂交（+）	治疗	随访时间/预后
1	Santucci等[5]	54	男	皮肤、中枢	NA	CD3、CD56、TIA-1、GrB、CD30、Ki-67、EBER	化疗	17个月/死亡
2	Merchant等[6]	37	男	肾（尸检）	-	CD3、CD8、TIA-1、EBER	未治疗	2周/死亡
3	Wu等[7]	41	男	皮肤	-	CD2、CD3、CD7、CD43、CD56、TIA-1、perforin、BCL2、EBER	化疗+造血干细胞移植	12个月/存活
4	Kuo等[8]	71	女	皮肤	-	CD3、CD56、TIA-1、Ki-67、EBER	未治疗	5个月/存活
5	Song等[9]	40	女	皮肤、中枢	-	LCA、CD3、CD56、GrB、TIA-1、Ki-67、EBER	化疗	7个月/存活
6	Cerroni等[1]	67	女	皮肤、中枢	NA	CD2、CD3、CD8、TIA-1、PCR-EBV	未治疗	1周/死亡
7	Cerroni等[1]	63	男	皮肤	NA	CD2、CD3、CD45RO、CD56、TIA-1、EBER	化疗	6个月/死亡
8	Nakamichi等[10]	23	女	皮肤、回肠？	-	CD3、CD56、TIA-1、EBER	化疗+造血干细胞移植	9个月/死亡
9	Liao等[11]	42	女	皮肤	-	CD3、CD56、GrB、Ki-67、EBER	化疗+放疗	14个月/存活
10	叶钦等[12]	64	女	皮肤、中枢？	NA	CD45、CD3、CD43、GrB、Ki-67、EBER	NA	NA/NA
11	蒋镭等[13]	68	女	皮肤	NA	CD3、CD56、GrB、Ki-67、EBER	化疗	2个月/死亡
12	蒋镭等[13]	22	男	皮肤	-	CD2、CD3、CD7、CD56、GrB、Ki-67、EBER	化疗	2个月/死亡
13	Liu等[14]	37	女	皮肤、中枢	-	CD3、CD56、GrB、Ki-67、EBER	化疗	13个月/死亡
14	Gebauer等[15]	72	男	皮肤、中枢	+	CD3、CD56、TIA-1、Ki-67、EBER	化疗	7个月/死亡
15	张立英等[16]	31	女	皮肤、鼻咽部？	+	CD3、CD56、GrB、Ki-67、EBER	化疗	失访/失访
16	Jang等[17]	23	女	皮肤	-	CD3、CD8、LCA、GrB、TIA-1、MPO、EBER	化疗+造血干细胞移植	>15个月/死亡
17	Xie等[18]	46	男	中枢、肾、前列腺、肝脏	-	CD3、CD8、GrB、Ki-67、EBER	未治疗	2个月/死亡
18	Alhumidi等[19]	48	女	皮肤	-	CD3、CD45、GrB、EBER	化疗	18个月/存活
19	Wang等[20]	45	男	皮肤	NA	CD2、CD3、GrB、TIA-1、perforin、CD56、EBER	未治疗	2周/死亡
20	Wang等[20]	52	女	皮肤、鼻咽部	NA	CD2、CD3、GrB、TIA-1、perforin、CD56、EBER	化疗	6个月/死亡
21	Wang等[20]	32	男	皮肤	NA	CD2、CD3、GrB、TIA-1、perforin、CD56、EBER	化疗	4个月/死亡
22	Wang等[20]	18	女	皮肤	-	CD2、CD3、GrB、TIA-1、perforin、CD56、EBER	化疗	3年/存活
23	Wang等[20]	51	男	皮肤	-	CD2、CD3、GrB、TIA-1、perforin、CD56、EBER	化疗	6个月/死亡
24	Bi等[21]	29	男	皮肤、肝脏	-	CD3、CD43、CD30、CD56、TIA-1、Ki-67、EBER	化疗	3个月/死亡
25~29	宋琳毅等[22]	38~83	男1例，女4例	均为皮肤	NA	均表达CD3、TIA-1、GrB、Ki-67、EBER，2例表达CD56、CD30	化疗（2例）；未治疗（3例）	1年/死亡；1年/死亡；1周/死亡；3个月/死亡；24个月/存活
30	莫祥兰等[23]	61	男	骨髓	+	CD3、CD8、GrB、TIA-1、Ki-67、EBER	化疗	4个月/死亡
31	Yang等[24]	67	男	附睾、皮肤	-	CD45、CD3、GrB、TIA-1、Ki-67、EBER	化疗	2个月/存活
32	焦霞等[25]	57	男	睾丸	NA	CD3、CD56、GrB、TIA-1、Ki-67、EBER	手术切除+化疗	22个月/存活
33	Yan等[26]	38	男	肺	-	CD2、CD3、CD56、GrB、TIA-1、Ki-67、EBER	化疗	2个月/死亡
34	Yan等[26]	21	男	肺	-	CD2、CD3、CD56、GrB、TIA-1、Ki-67、EBER	未治疗	2个月/死亡
35	Yan等[26]	23	男	皮肤	+	CD3、CD56、GrB、TIA-1、Ki-67、EBER	化疗	18个月/死亡
36	Yan等[26]	54	女	皮肤	-	CD3、CD56、GrB、TIA-1、Ki-67、EBER	未治疗	3个月/死亡
37	Alegría-Landa等[27]	81	男	皮肤	-	CD3、CD30、GrB、perforin、EBER	未治疗	2周/死亡
38	Okonkwo等[28]	51	男	皮肤、中枢？	NA	CD3、CD8、GrB、TIA-1、EBER	NA	NA/NA
39	蔡兆根等[29]	47	女	皮肤	NA	CD3、CD43、CD56、TIA-1、Ki-67、EBER	化疗	3个月/存活
40	张立英等[30]	18	男	淋巴结（多发）、鼻咽部、直肠？	-	LCA、CD3、CD7、CD56、GrB、TIA-1、Ki-67、EBER	化疗+放疗+造血干细胞移植	65个月/存活
41	Elshiekh等[31]	72	男	肾	-	CD3、CD56、MUM1、MYC、GrB、Ki-67、EBER	NA	3周/死亡
42	Zanelli等[32]	54	男	心、肺、肾、脾、肝、脑（尸检）	+	CD2、CD3、CD56、GrB、perforin、Ki-67、EBER	未治疗	3d/死亡
43	何靖等[33]	35	女	皮肤	-	CD3、CD56、GrB、TIA-1、Ki-67、EBER	化疗	6个月/死亡
44	Wang等[34]	60	男	皮肤、淋巴结？	-	CD3、CD56、GrB、TIA-1、Ki-67、EBER	化疗	18个月/存活
45	Yu等[35]	40	男	皮肤	NA	CD3、CD7、CD56、GrB、TIA-1、EBER	NA	NA/NA
46	Fujikura等[36]	20	男	肺	NA	CD3、CD4、CD45、CD56、GrB、perforin、Ki-67、EBER	化疗+造血干细胞移植	18个月/存活
47	Fujikura等[36]	65	男	回肠、肝、脾、皮肤（尸检）	NA	CD3、CD5、CD7、CD8、CD45、GrB、perforin、Ki-67、EBER	未治疗	1d/死亡

注：NA：未提供

**表2:** 8例ALK阴性或ALK阳性血管内间变性大细胞淋巴瘤（IVALCL）患者的临床病理资料

例号	文献来源	年龄（岁）	性别	诊断	受累部位	骨髓受累	免疫组化	EBER	治疗	随访/预后
1	Takahashi等[37]	72	男	ALK^−^ IVALCL	肝、脾、肾、肺、淋巴结（尸检）	+	CD2、CD4、CD43、CD45RO、CD30、GrB、TIA-1、perforin均（+），ALK、EMA均（−）	−	化疗	1周/死亡
2	Rieger等[38]	79	男	ALK^−^ IVALCL	皮肤+淋巴结	−	CD2、CD4、CD43、BCL2、CD30均（+），ALK、EMA、细胞毒性分子均（−）	NA	化疗	NA/NA
3	Zizi-Sermpetzoglou等[39]	48	女	ALK^−^ IVALCL	皮肤	NA	CD45、CD3、CD4、CD30均（+），ALK（−），其余未获得	NA	NA	NA/NA
4	Wang等[40]	47	女	ALK^−^ IVALCL	皮肤	未做	CD45、CD3、CD4、CD5、CD30均（+），ALK、EMA、细胞毒性分子均（−）	NA	化疗	8年/存活，后失访
5	Iacobelli等[41]	39	女	ALK^−^ IVALCL	皮肤	−	CD2、CD4、CD7、CD30均（+），ALK、EMA、细胞毒性分子均（−）	−	化疗+放疗	7个月/存活
6	Metcalf等[42]	83	男	ALK^−^ IVALCL	皮肤	未做	CD2、CD3、CD4、CD43、CD30均（+），ALK、细胞毒性分子均（−）	−	放疗	48个月/存活
7	Krishnan等[43]	33	女	ALK^+^ IVALCL	皮肤+淋巴结	NA	CD30、ALK均（+），余未提供	NA	化疗	NA/NA
8	徐德等[44]	41	女	ALK^+^ IVALCL	中枢	−	LCA、CD3、ALK、EMA、GrB均（+），CD30（−）	NA	手术	8个月/死亡

注：EBER：EB病毒编码小RNA；NA：未提供

## 讨论

血管内淋巴瘤属于非霍奇金淋巴瘤的少见类型，1959年由Pfleger和Tappeiner首次报道为亲血管内皮细胞瘤[45]。随着免疫组织化学及分子学的发展，逐渐认识到这类疾病是起源于淋巴造血系统的恶性肿瘤，以B细胞来源为主（占85％～90％）。WHO 2001年版淋巴造血系统肿瘤分类将血管内B细胞淋巴瘤（B-IVL）定义为结外弥漫大B细胞淋巴瘤（DLBCL）的一个罕见亚型，另有少数为T细胞和NK细胞来源（占10％～15％）。自2003年Santucci等[5]报道首例血管内NK/T细胞淋巴瘤（IVNKTL）以来，关于血管内外周T细胞和NK细胞淋巴瘤的报道逐渐增多，但在最新的WHO 2017年版分类中尚未作为独立类型，仅在血管内大B细胞淋巴瘤章节中予以简单叙述[46]。

血管内T细胞和NK细胞淋巴瘤是一类局限生长于血管内的高度侵袭性疾病，发病年龄范围较广，有1例发生于死产胎儿[47]，年龄最大者87岁[1]，主要为中老年人，其发病机制尚不清楚，多数伴有EBV感染。多表现为发热、全身乏力、体重减轻、盗汗、贫血和血清乳酸脱氢酶水平升高等[1],[6],[22]。骨髓受累可见，也可见多器官受累病例[32],[36]–[37]。此类疾病最常受累的器官是皮肤及中枢神经系统，其他部位如肺、脾、肾、肝、肠、卵巢、宫颈、睾丸等均可发生。目前累及淋巴结的病例报道少见，1例为发生于淋巴结内的血管内NK/T细胞淋巴瘤病例，但PET-CT显示鼻咽部及直肠考虑恶性肿瘤，遗憾的是此例患者未做鼻腔及直肠的病理活检，未能证实是原发于淋巴结的IVNKTL还是原发于鼻腔或肠道的结外鼻型NK/T细胞淋巴瘤伴淋巴结血管内播散[30]；第2例是原发于右侧腹股沟淋巴结的血管内T细胞淋巴瘤[2]，此病例免疫组化表型及分子检测均不完善，不能明确分型；第3、4例为原发于皮肤的血管内ALCL累及淋巴结[38],[43]；第5例为尸检报告ALKIVALCL累及淋巴结[37]。尚有数例患者影像学回报淋巴结肿大，可疑受累，但无病理活检证实[1],[34]。其余均无淋巴结受累的报道。本例患者PET-CT示除全身多发淋巴结肿大外，尚有脾肿大，均考虑恶性淋巴增殖性疾病，遗憾的是此患者未做脾病理活检。因此本例是为数不多的淋巴结受累的血管内T细胞淋巴瘤，也是第1例明确淋巴结受累的IVPTCL，NOS报道。

血管内T细胞和NK细胞淋巴瘤组织病理学特点为中等偏大的肿瘤细胞局限生长于血管腔内，细胞中等大小，细胞核呈圆形、卵圆形或不规则形，可见核仁，核分裂象易见，部分病例可见血管内纤维素样渗出物或血栓形成，均表达部分T细胞或NK细胞标记，多数原位杂交EBV-EBER阳性，表达一种或多种细胞毒性标志物（TIA-1、Granzyme B和穿孔素），Ki-67增殖指数均较高，说明此类疾病的高增殖活性。而IVALCL细胞异型性明显，核深染呈卵圆形或不规则形，略偏向一侧，可见马蹄形或肾形核，表达CD30及部分T细胞标志，ALK（+/−），可表达细胞毒性分子标志。此类疾病应与上皮、间叶及生殖细胞等恶性肿瘤发生脉管内癌栓、血管内大B细胞淋巴瘤及其他淋巴瘤伴脉管内播散等疾病相鉴别。前两者可通过免疫组化CK、Vim、B细胞标志等鉴别，肿瘤主要发生在实质脏器或组织内，而血管内T细胞和NK细胞淋巴瘤主要发生于血管内。

目前针对此类疾病多采用CHOP方案（环磷酰胺+阿霉素+长春新碱+地塞米松）或改进的方案化疗。Yan等[26]总结29例IVNKTL患者的生存分析，结果显示B症状和接受化疗与预后相关。有多脏器受累或无皮肤表现患者的临床疗效较有单纯皮肤表现的患者差。5例患者采用化疗联合造血干细胞移植治疗[7],[10],[17],[30],[36]，预后均较好，因病例数太少，仍需更多病例进一步评估。本例报道的患者预后较差，尽管给予积极的干预治疗，但仍于确诊后2个月内死亡。因患者有心脏疾病病史，化疗药物对心脏有毒性作用，故不能客观地评价及分析预后。

原发于血管内的外周T细胞和NK细胞淋巴瘤发病过程均呈高度侵袭性，目前尚无有效的治疗方案。典型病例的病理诊断并不十分困难，但对于肿瘤细胞较少及较局限生长于血管腔内的病变容易漏诊、误诊，快速、精确诊断仍具有一定挑战。目前对本病尚缺乏足够的认识，其发病机制、有效的治疗方案及预后相关因素均需积累更多病例进一步研究。
